# The Effectiveness of Vitamin D Intake in Improving Symptoms and Relapses of Multiple Sclerosis: A Systematic Review

**DOI:** 10.7759/cureus.68565

**Published:** 2024-09-03

**Authors:** Abhishek Gill, Chijioke Orji, Maiss Reghefaoui, Tariladei S Peresuodei, Priyanka Thota, Michell Susan Saavedra Palacios, Ana P Arcia Franchini

**Affiliations:** 1 Internal Medicine, Christian Medical College & Hospital, Ludhiana, IND; 2 Internal Medicine and Neurology, California Institute of Behavioral Neurosciences & Psychology, Fairfield, USA; 3 Orthopedics, California Institute of Behavioral Neurosciences & Psychology, Fairfield, USA; 4 Internal Medicine, University of Debrecen, Debrecen, HUN; 5 Internal Medicine, California Institute of Behavioral Neurosciences & Psychology, Fairfield, USA; 6 College of Medicine, Siddhartha Medical College, Vijayawada, IND; 7 College of Medicine, University of Cuenca, Cuenca, ECU; 8 Psychiatry and Behavioral Sciences, California Institute of Behavioral Neurosciences & Psychology, Fairfield, USA

**Keywords:** systematic review, edss, relapse, vitamin d, multiple sclerosis

## Abstract

There are disagreements over the effectiveness of vitamin D supplementation in treating multiple sclerosis (MS) patients' symptoms and reducing relapses. The goal of this systematic review is to assess the effect of vitamin D supplements on improving symptoms and relapses in MS patients. Following the Preferred Reporting Items for Systematic Reviews and Meta-Analyses (PRISMA) guidelines, a systematic review was conducted by searching eight databases: Embase, PubMed, Cochrane Library, MEDLINE, CINAHL, Web of Science, Scopus, and Google Scholar. The RoB 2 tool was used to evaluate the quality of the studies that were included in the analysis. From the 1,345 studies identified, 16 randomized controlled trials were selected. All studies reported that vitamin D administration significantly increased the mean serum 25(OH)D compared with the placebo group. Also, most included studies revealed a significant improvement in magnetic resonance imaging (MRI) brain lesion markers. However, most studies showed that being treated with vitamin D instead of placebo showed no significant effect on relapse rates, fatigue, Expanded Disability Status Scale (EDSS), serum neurofilament light chain (NfL), calcium, and cytokine levels, except for quality-of-life transforming growth factor beta (TGF-β). This systematic review shows that the effect of vitamin D supplements on improving symptoms and relapses during treatment in MS patients remains inconclusive.

## Introduction and background

Multiple sclerosis (MS) is a central nervous system (CNS) illness that is persistent, degenerative, and demyelinating [1]. MS affects young individuals and is frequently linked to a serious disability and a lower quality of life [2]. A wide range of symptoms, including motor or sensory impairment or visual problems, are brought on by localized inflammation in the CNS, which disrupts the signaling of the afflicted neurons. Fatigue and depression are additional MS symptoms that cannot be fully explained by local inflammation. The most common MS symptom described is fatigue, experienced by up to 90% of MS patients [3]. An autoimmune inflammatory reaction mediated by several immune system components appears to be the main mechanism of this disease [4]. Although the origin and pathogenesis of MS are unknown, genetic and environmental factors play a role in the disease [5]. MS is frequently characterized by disability and high morbidity. Therefore, symptom management must be a long-term concern because disease-modifying medications only halt the disease's course [6].

According to numerous research, vitamin D status may be a non-genetic component that influences the onset of MS [7]. The first source of support comes from ecological studies demonstrating a negative correlation between ambient UV-B radiation levels, which are necessary for producing vitamin D, and MS prevalence or mortality [8]. It was also shown that 50% to 70% of patients in various MS populations had low blood levels of 25(OH)D [9]. Soilu-Hanninen et al. observed a significant association between serum levels of 25(OH)D and MS in a cohort of newly diagnosed Finnish MS patients. Compared to the control group, the MS patients exhibited considerably lower serum levels of 25(OH)D [10]. It has also been demonstrated that 25(OH)D levels correlate with the severity of MS. However, it has been observed that individuals with relapsing-remitting MS may also have lower levels of 25(OH)D. [10-11]. Additionally, research has revealed that patients with MS tend to exhibit lower levels of vitamin D during relapse as compared to periods of remission [12-13]. The most convincing evidence establishing an association between vitamin D and MS activity and progression is derived from a prior analysis that investigated vitamin D levels to predict early MS activity and progression within the group of patients participating in the BENEFIT study [14].

However, it is still unclear exactly how vitamin D may be associated with MS. Vitamin D exhibits anti-inflammatory and immunomodulatory qualities [13]. The activated form of vitamin D, also known as calcitriol or 1,25-dihydroxycholecalciferol (1,25(OH)2D), is involved in many biological functions, most of which are mediated through vitamin D receptors (VDRs). Most immune system cell types, including monocytes, antigen-presenting cells, and activated lymphocytes, express this receptor [15]. The generation of TH1 and TH2 cytokines and lymphocyte proliferation are altered by VDR activation. Vitamin D decreases the synthesis of cytokines related to inflammation and MS and blocks the growth of B and T lymphocytes [16]. According to a previous study, 39 MS patients who were administered a daily dose of 25 g of vitamin D for six months experienced a rise in plasma 25(OH)D levels. Moreover, there was a noteworthy elevation in the cytokine transforming growth factor beta (TGF-β), and a reduction in IL-2 mRNA levels was also observed [17]. Moreover, it was reported that the incidence of gadolinium-enhancing lesions on magnetic resonance imaging (MRI) decreased in 12 patients supplemented with up to 1,000 g/day of vitamin D for 28 weeks [18].

This review aims to analyze the available data on how vitamin D supplementation, compared to a placebo, affects symptom reduction in individuals with MS. The evaluation covers improvements in serum 25(OH)D levels, fatigue, relapse rates, quality of life, EDSS (Expanded Disability Status Scale) scores, serum neurofilament light chain (NfL) MRI brain lesion markers, cytokine levels, and calcium levels.

## Review

Methodology

Design

This systematic review follows the Preferred Reporting Items for Systemic Reviews and Meta-Analyses (PRISMA) 2020 guidelines [19]. This review did not require review or approval by the ethics committee.

Search Strategy

A literature search was conducted on Embase, PubMed, Cochrane Library, MEDLINE, CINAHL, Web of Science, Scopus, and Google Scholar from database inception until July 2023 to search for articles that may be eligible. A search strategy was employed using specific key search terms, which included “Multiple Sclerosis” OR “MS” AND “vitamin D supplementation” OR “vitamin D” OR “cholecalciferol” OR “ergocalciferol” OR “alfacalcidol” OR “calcitriol.”

Selection Criteria

The articles were selected in two steps: first, the title and abstract were screened and then the complete text. Two impartial reviewers carried out the search and screening procedure. After eliminating duplicate studies, relevant articles were carefully examined based on their title and abstracts. To be considered, studies needed to focus on the clinical efficacy of vitamin D supplementation in managing symptoms of people with MS. Any disagreements were resolved by inviting a third author to participate in the discussion. The effectiveness was measured using different parameters such as serum 25(OH)D, relapse rates, disability status by EDSS scores, cytokine profile, quality of life, NfL, T2 lesion load, and new T2 or T1 Gd enhancing lesions. The other studies were then thoroughly reviewed by analyzing their complete texts to ensure they met the eligibility criteria.

Inclusion criteria for articles were as follows: (1) randomized controlled trials (RCT) with an intervention and control group, (2) a clinical diagnosis of MS, and (3) the main outcome assessments encompassing serum 25(OH)D levels, disability status determined through EDSS scores, relapse rates, quality of life, cytokine profile, NfL T2 lesion load, and the presence of new T2 or T1 Gd-enhancing lesions. Exclusion criteria comprised the following: (1) unavailability of electronically accessible full text, (2) publication not in the English language, and (3) RCT on animals.

Data Extraction

Two reviewers performed data extraction on each chosen article using a standardized form. The following information was extracted and entered into a Microsoft Excel spreadsheet: study ID (name of first author, year of publication), country, study design, study population, gender (male/female), mean age, intervention, duration, and outcome measures.

Quality Assessment

Version 2 of the Cochrane risk-of-bias tool for randomized trials (RoB 2) was used to assess the risk of bias for RCTs [20]. Bias is evaluated as a judgment (high, low, or some concerns) for individual elements from five domains (bias arising from the randomization process, bias due to deviations from intended intervention, bias due to missing outcome data, bias in measurement of the outcome, and bias in selection of the reported results). After two reviewers independently evaluated each item, the disagreed items were reanalyzed. The evaluation results were summarized online using the Robvis website [21].

Results

Search Results

**Figure 1:**
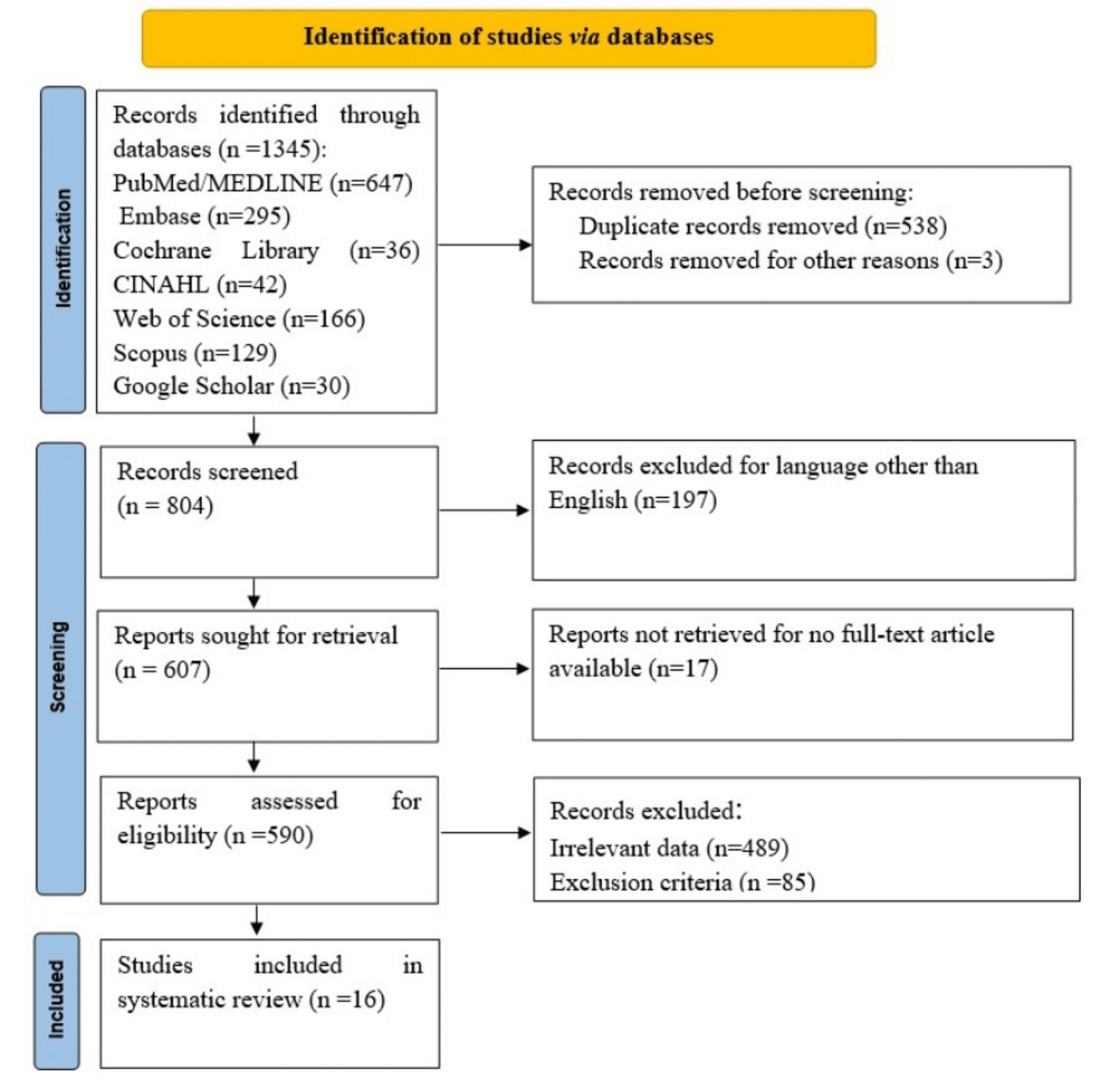
PRISMA flow diagram PRISMA, Preferred Reporting Items for Systematic Reviews and Meta-Analyses

Figure 1 depicts information regarding the methods employed for literature search and screening. Initially, 1,345 articles were retrieved through the literature search. Following eliminating duplicates, 804 articles underwent screening at the title/abstract level, with 590 proceeding to full-text review. Subsequently, 574 studies were excluded during the full-text assessment, leaving 16 studies that conformed to the predetermined criteria and were consequently incorporated into this systematic review.

Study Characteristics

Table 1 provides an overview of the key characteristics of the studies encompassed in this analysis. These studies were published within the timeframe spanning from 2011 to 2020 and distributed among the following nine countries: Finland (n=4), Iran (n=4), Norway (n=4), the Netherlands (n=2), Israel (n=1), and France (n=1). Sample sizes ranged from 30 to 181 participants. The majority of participants were women. The mean age of participants ranged from 31.50 to 41.3 years. The mean disease duration varied between two and 11 years. The duration of treatment ranged between three and 24 months (Table 1).

**Table 1:** Characteristics of included articles MS, multiple sclerosis; RRMS, relapsing-remitting multiple sclerosis

Study ID	Country	Study design	Study population	Intervention and control groups	Gender (M/F)	Mean age (years)	Mean disease duration (years)	Duration
Achiron et al. 2015 [22]	Israel	Randomized, double-blind, placebo-controlled study	158 MS patients with significant fatigue	Vitamin D (1 mcg/d, N=80) and placebo (N=78)	Vitamin D: 21/59 and placebo: 19/59	Vitamin D: 41.3 ± 9.8 and placebo: 40.8 ± 8.7	6.2 ± 5.5	6 months
Åivo et al. 2012 [23]	Finland	Double-blind, randomized, parallel-group one-year multicenter trial	30 RRMS patients	Vitamin D (20,000 IU/week, N=15) and placebo (N=15)	Vitamin D: 6/9 and placebo: 6/9	Vitamin D: 37.0 (25.0–53.0) and placebo: 32.0 (22.0–47.0)	Vitamin D: 3.0 (0.6–15.2) and placebo: 1.5 (0.3–4.7)	12 months
Åivo et al. 2015 [24]	Finland	Double-blind, randomized, parallel-group one-year multicenter trial	59 RRMS patients	Vitamin D (20,000 IU/week, N=30) and placebo (N=29)	Vitamin D: 12/18 and placebo: 10/19	Vitamin D: 38.0 (22.0-53.0) and placebo: 35.0 (24.0–53.0)	Vitamin D: 3.0 (0.5–21.0) and placebo: 2.0 (0.2-15.0)	12 months
Ashtari et al. 2015 [25]	Iran	Randomized, double-blind, placebo-controlled clinical trial	89 RRMS patients	Vitamin D (50,000 IU every five days, N=44) and placebo (N=45)	Vitamin D: 9/35 and placebo: 5/40	Vitamin D: 31.50 ± 7.60 and placebo: 34.60 ± 10.12	Vitamin D: 4.10 ± 3.73 and placebo: 4.46 ± 3.99	3 months
Camu et al. 2019 [26]	France	Double-blind, placebo-controlled parallel-group, two-year study	181 RRMS patients	Vitamin D (100,000 IU every other week for 96 weeks, N=63) and placebo (N=66)	Vitamin D: 13/50 and placebo:27/39	Vitamin D:38.40 ±9.31 and placebo: 36.73 ±8.37	Vitamin D:5.13 ±5.33 and placebo: 5.59 ±4.83	24 months
Hänninen et al. 2020 [27]	Finland	Randomized controlled trial	66 RRMS patients	Vitamin D (20,000 IU, N=17) and placebo (N=15)	Not disclosed	Vitamin D: 38.3 ± 8.17 and placebo: 38.5 ± 7.32	Vitamin D:7.79 ±6.94 and placebo: 7.31 ±5.92	12 months
Holmøy et al. 2019 [28]	Norway	Placebo-controlled randomized study	68 RRMS patients	Vitamin D (20,000 IU weekly, N=35) and placebo (N=33)	Vitamin D: 11/24 and placebo: 9/24	Vitamin D: 40.0 ± 8.0 and placebo: 41.0 ± 6.0	Vitamin D: 11.0 ± 7.0 and placebo: 10.0 ± 7.0	24 months
Kampman et al. 2012 [29]	Norway	Randomized controlled trial	68 patients with MS	Vitamin D (20,000 IU weekly, N=35) and placebo (N=33)	Vitamin D: 11/24 and placebo: 9/24	Vitamin D: 40.0 and placebo: 41.0	Vitamin D: 11.0 and placebo: 10.0	24 months
Mosayebi et al. 2011 [30]	Iran	Prospective randomized controlled trial	62 MS patients	Vitamin D (300,000 IU/month, N=26) and placebo (N=33)	Vitamin D: 9/17 and placebo: 9/25	Vitamin D: 37.0 ± 9.0 and placebo: 35.0 ± 9.0	Vitamin D: 4.15± 3.3 and placebo: 6.4 ± 4.6	6 months
Rolf et al. 2017 [31]	Netherlands	Randomized pilot study	40 RRMS patients	Vitamin D (14.000 IU/day, N=20) and placebo (N=20)	Vitamin D: 6/14 and placebo: 8/12	Vitamin D: 38.5 ± 7.8 and placebo: 37.6 ± 9.6	Vitamin D: 7.5 and placebo: 5.7	12 months
Røsjø et al. 2015 [32]	Norway	Double-blinded randomized placebo-controlled trial	68 RRMS patients	Vitamin D (20.000 IU/week, N=36) and placebo (N=32)	Vitamin D: 11/25 and placebo: 9/23	Vitamin D: 40.0 and placebo: 41.0	Vitamin D: 11.0 and placebo: 10.0	24 months
Shaygannejad et al. 2012 [33]	Iran	Phase II double-blind placebo-controlled randomized clinical trial	50 RRMS patients	Vitamin D (0.5 μg/day, N=25) and placebo (N=25)	Vitamin D: 3/22 and placebo: 3/22	Vitamin D: 38.6 ±8.4 and placebo: 37.9 ±7.9	Vitamin D: 4.5 ±2.7 and placebo: 4.1 ±1.7	12 months
Smolders et al. 2020 [34]	Netherlands	Double-blind, placebo-controlled and randomized design,	40 interferon beta-treated RRMS patients	Vitamin D (14.000 IU/day, N=24) and placebo (N=16)	Vitamin D: 7/17 and placebo: 5/11	Vitamin D: 37.0 and placebo: 40.0	Vitamin D: 6.5 and placebo: 5.7	12 months
Soilu-Hanninen et al. 2012 [35]	Finland	Randomized, double-blind, placebo-controlled trial	66 MS patients	Vitamin D (20 000 IU, N=34) and placebo (N=32)	Vitamin D: 13/21 and placebo: 12/20	Vitamin D: 39.0 and placebo: 35.0	Vitamin D: 3.0 and placebo: 2.4	12 months
Steffensen et al. 2011 [36]	Norway	Single-center, balanced randomized, double-blinded, placebo-controlled, parallel-group	68 MS patients	Vitamin D (20.000 IU/week, N=35) and placebo (N=33)	Vitamin D: 11/24 and placebo: 9/24	Vitamin D:39.7 and placebo: 41.0	Vitamin D: 10.9 and placebo: 10.0	24 months
Toghianifar et al. 2015 [37]	Iran	Randomized, double-blind, placebo-controlled clinical trial	89 RRMS patients	Vitamin D (50.000 IU every five days, N=44) and placebo (N=45)	Vitamin D: 9/35 and placebo: 5/40	Vitamin D:31.50 ± 7.60 and placebo: 34.60 ± 10.12	Vitamin D: 4.10 ± 3.73 and placebo: 4.46 ± 3.99	3 months

Quality Assessment

**Figure 2:**
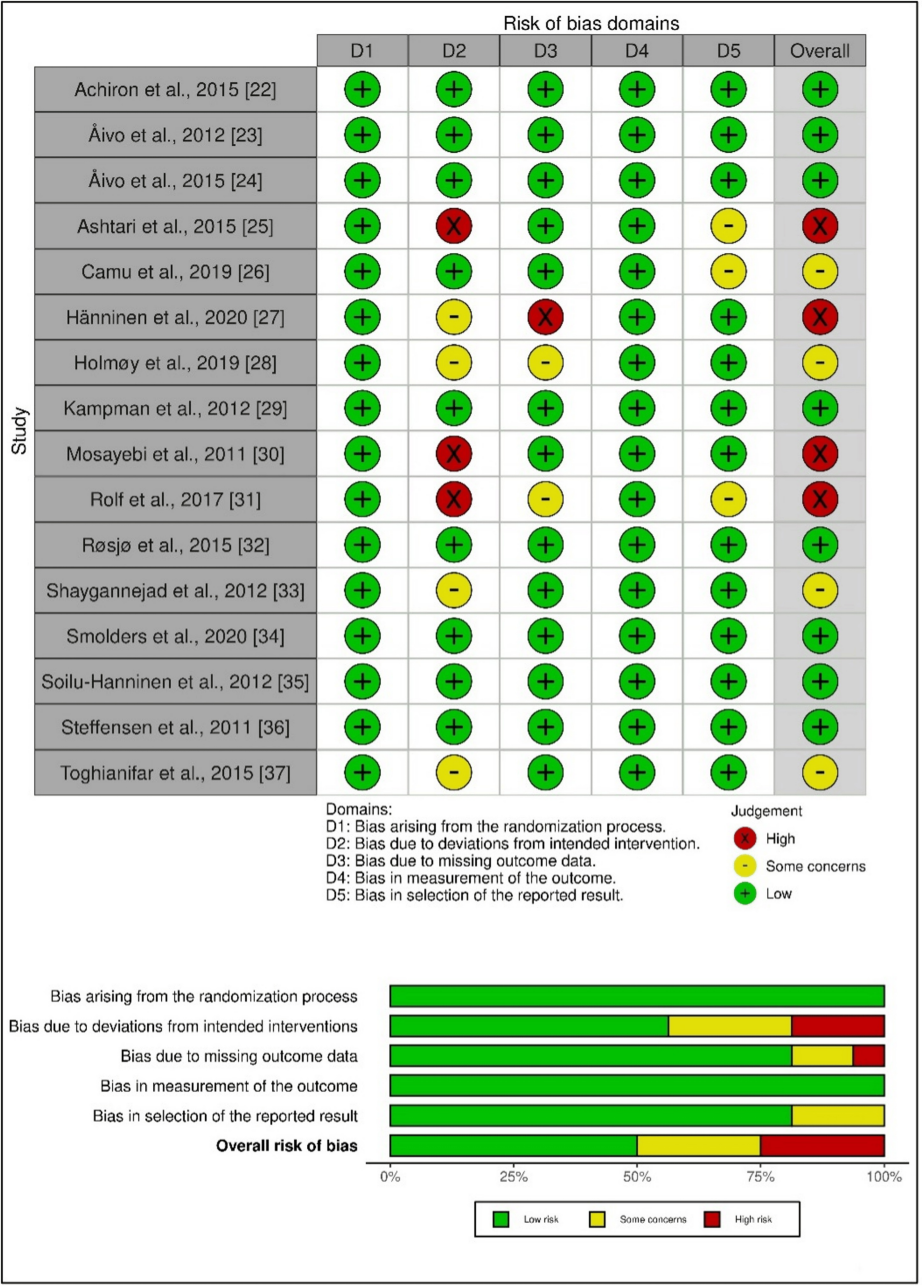
Risk-of-bias evaluation of the included studies using the ROB 2 tool

Among the included studies, four (25%) of 16 studies were regarded as having a high risk of bias, and also four (25%) of 16 were rated as having some concerns. However, 50% of studies presented a low risk of bias (Figure 2).

Key findings

Many outcomes were investigated among the included articles, such as serum 25(OH)D, EDSS, relapse rate, cytokine levels, fatigue, and quality of life (Table 2).

**Table 2:** Main findings reported by the included articles ARR, annual relapse rate; BMD, bone mineral density; BOD, burden of disease; EDSS, expanded disability status scale; FIS, fatigue impact scale; FSS, fatigue severity scale; HADS-D, Hospital Anxiety and Depression Scale – Depression; IL, interleukin; INF-γ, interferon‐gamma; MRI, magnetic resonance imaging; MS, multiple sclerosis; MSFC, multiple sclerosis functional composite; NfL, neurofilament light chain; QoL, quality of life; serum 25(OH), serum 25-hydroxyvitamin D; SI, stimulation index; TGF-β, transforming growth factor-beta; TNF-α, tumor necrosis factor-alpha

Study	Outcomes	Findings
Achiron et al. 2015 [22]	MS-related fatigue (FIS), QoL, and relapse rate	Alfacalcidol decreased the mean relative FIS score as compared with placebo (p=0.007). QoL improved in alfacalcidol-treated patients as compared with placebo in the RAYS psychological (p=0.033) and social (p=0.043) sub-scales. The alfacalcidol-treated group had a reduced number of relapses (p=0.006) and a higher proportion of relapse-free patients (p=0.007).
Åivo et al. 2012 [23]	EDSS, ARR, T1-enhancing lesions, new or enlarging T2/PD lesions, MRI activity, timed 10-foot tandem walk and timed 25-foot tandem walk	There was not a statistically significant difference between vitamin D and placebo groups in terms of EDSS, ARR, timed 10-foot tandem walk and 25-foot tandem walk. There was a statistically significant reduction in the number of T1-enhancing lesions, a smaller T2 lesion volume growth, and fewer new/enlarging T2 brain MRI lesions in the vitamin D3-treated subgroup than in the placebo-treated subgroup patients.
Åivo et al. 2015 [24]	Cytokine levels: TGF-β, INF-γ, IL-17A, IL-2, IL-10, IL-9, IL-22, IL-6, IL-13, IL-4, IL-5, IL-1β, and TNF-α	TGF-β levels significantly increased in the vitamin D-treated group compared to placebo (p=0.024). There were no significant changes observed in the levels of the other cytokines in either group.
Ashtari et al. 2015 [25]	IL-10, calcium, and vitamin D	Serum levels of IL-10 did not differ significantly in vitamin D and placebo groups (p = 0.158). Serum levels of calcium did not differ significantly in vitamin D and placebo groups (p = 0.302). Vitamin D levels showed a significant difference in vitamin D and placebo groups (p < 0.001).
Camu et al. 2019 [26]	Serum 25(OH)D, ARR, MRI brain lesion markers, EDSS, and QoL (EQ-5D-3L or the PASAT-3 total score)	Vitamin D administration roughly tripled mean serum 25(OH)D compared with the placebo group (p < 0.001). All efficacy parameters favored cholecalciferol with an ARR reduction (p = 0.012), fewer new hypointense T1-weighted lesions (p = 0.025), a lower volume of hypointense T1-weighted lesions (p = 0.031), and a lower progression of EDSS (p =0.026). There was no difference in the change in QoL measured by the EQ-5D-3L or the PASAT-3 total score between the two experimental groups.
Hänninen et al. 2020 [27]	NfL levels	Serum NfL levels were similarly low in patients supplemented with high-dose vitamin D or placebo.
Holmøy et al. 2019 [28]	NfL levels	Compared to placebo, vitamin D supplementation had no overall effect on the change in serum levels of NfL (p=0.93 at week 48 and p=0.56 at week 96).
Kampman et al. 2012 [29]	Serum 25(OH)D, ARR, EDSS, MSFC components, grip strength, FSS	Vitamin D administration significantly increased the mean serum 25(OH)D compared with the placebo group (p < 0.001). After 96 weeks, there was no statistically significant difference observed between the groups in terms of ARR (p = 0.25), EDSS (p = 0.97), MSFC components, grip strength, or FSS.
Mosayebi et al. 2011 [30]	Serum 25(OH)D, gadolinium-enhancing lesions, EDSS, the levels of cell proliferation (stimulation index, SI), and cytokine levels (IFN-γ, IL-10, TGF-β)	Vitamin D administration significantly increased the mean serum 25(OH)D compared with the placebo group (p < 0.0001). There was no significant difference between the treatment and the control groups in the EDSS and several gadolinium-enhancing lesions. The SI in the vitamin D treatment group was significantly lower than that in the control group (p= 0.001). The levels of IFN-γ were unaffected by vitamin D. The levels of TGF-β and IL-10 in the vitamin D treatment group were significantly higher than that in the control group (p = 0.0001)
Rolf et al. 2017 [31]	Serum 25(OH)D, HADS-D, FSS, cytokine levels (TNF-α, IL-10)	A significantly larger difference in the serum 25(OH)D between treatment and placebo groups was detected (p<0.01). No significantly different reductions were detected between groups in terms of HADS-D (p=0.78) and FSS (p=0.95). No significant reductions in TNF-α (p=0.37) and IL-10 (p=0.54) were found in the vitamin D3 compared to the placebo arm.
Røsjø et al. 2015 [32]	Serum 25(OH)D and inflammatory markers	A significantly larger difference in the serum 25(OH)D between treatment and placebo groups was detected (p<0.001). No significant differences appeared between the vitamin D group and the placebo group for any of the inflammation markers.
Shaygannejad et al. 2012 [33]	EDSS and relapses	There were no significant differences in the EDSS or number of relapses at the end of the study period between the vitamin D and placebo groups.
Smolders et al. 2020 [34]	Serum 25(OH)D and NfL levels	Serum 25(OH)D levels were significantly increased in the vitamin D group when compared to the placebo group (p<0.01). NfL levels did not differ between vitamin D and placebo groups (P=0.74).
Soilu-Hanninen et al. 2012 [35]	Serum 25(OH)D, T2 BOD, number of MRI enhancing T1 lesions and new T2 lesions, ARR, and EDSS	Serum 25(OH)D levels were significantly increased in the vitamin D group when compared to the placebo group (p<0.001). There was a significantly lower number of T1-enhancing lesions in the vitamin D group when compared to the placebo group (p=0.004). There were no significant differences in the T2 BOD and number of new T2 lesions between both groups. There were no significant differences in the ARRs. There was no significant difference in EDSS between groups.
Steffensen et al. 2011 [36]	BMD	Percentage change in BMD did not differ between groups at any site.
Toghianifar et al. 2015 [37]	Serum 25(OH)D, calcium, and IL-17	Serum 25(OH)D levels were significantly increased in the vitamin D group when compared to the placebo group (p<0.001). There were no significant differences in the calcium (p=0.302) and IL-17 (p=0.960) levels between groups.

Serum 25(OH)D

Nine studies compared serum 25(OH)D levels between treatment and control groups [25,26,29-32,34,35,37]. All studies confirmed that vitamin D administration significantly increased the mean serum 25(OH)D compared with the placebo group.

Fatigue

Three studies investigated the improvement of fatigue after the administration of vitamin D in comparison with the placebo group. Achiron et al. revealed that vitamin D administration significantly decreased fatigue as measured by the fatigue impact scale compared with placebo (p=0.007) [22]. However, Rolf et al. and Kampman et al. did not report a significant difference using the fatigue severity scale (FSS) (p=0.95) [29,31].

Quality of Life

Two studies investigated the improvement of quality of life after the administration of vitamin D in comparison with the placebo group. Achiron et al. revealed that vitamin D administration significantly increased quality of life as measured by RAYS psychological and social sub-scales compared with placebo (p=0.007) [22]. However, there was no difference in the change in the quality of life measured by the EQ-5D- 3L or the PASAT-3 total score between the two experimental groups, as described by Camu et al. [26].

Relapse Rate

Six studies reported relapse rates/annual relapse rates after the administration of vitamin D in comparison with the placebo group. The majority of studies (4/6) did not find a statistically significant difference between the relapse rates of the vitamin D group and the placebo group. [23,29,33,35], while two studies revealed that vitamin D-treated group had a significantly reduced number of relapses compared to placebo [22,26].

EDSS

Six studies reported improvement of the EDSS after the administration of vitamin D in comparison with the placebo group. The majority of studies (5/6) did not find a statistically significant difference between vitamin D and placebo groups in terms of EDSS [23,29,30,33,35], while Camu et al. revealed that the vitamin D- treated group had a significantly lower progression of EDSS compared to placebo [22,26].

MRI Brain Lesion Markers

Four studies reported improvement of MRI lesions after the administration of vitamin D in comparison with the placebo group. Three studies showed a statistically significant reduction in the number of T1- or T2-enhancing lesions in the vitamin D3-treated subgroup than in the placebo-treated subgroup patients [23,26,35]. However, Mosayebi et al. reported no significant difference between the treatment and the control groups in terms of the number of gadolinium-enhancing lesions [30].

Serum NfL Levels

Three studies compared the serum NfL levels between vitamin D3-treated and placebo-treated patients. All studies demonstrated that vitamin D supplementation had no overall effect on the change in serum levels of NFL compared to placebo [27,28,34].

Cytokine Levels

The levels of TGF-β in the vitamin D treatment group were significantly higher than the control group, as reported by Aivo et al. and Mosayebi et al. [24,30]. However, no significant difference was detected between groups for the other cytokines (IL-10, IL-17, INF-γ) [24,25,30,37].

Calcium Levels

Two studies compared calcium levels between vitamin D3-treated and placebo-treated patients. Both studies revealed that serum levels of calcium did not differ significantly in vitamin D and placebo groups [25,37].

Also, it was shown that there were no significant differences between vitamin D and placebo groups for depression (Hospital Anxiety and Depression Scale - Depression [HADS-D]) [31], inflammation markers [32], and bone mineral density [36].

Discussion

Involvement of vitamin D in a range of chronic illnesses, including autoimmune diseases such as MS, systemic lupus erythematosus, and psoriasis, has been shown by recent studies. These autoimmune disorders and levels of vitamin D 1,25 were found to have a high positive correlation. These results gave rise to the theory that high vitamin D levels may lessen the likelihood of MS relapses and disease progression [38].

One of the most prevalent signs of MS is fatigue, which is linked to a lower quality of life. We still have a startling lack of understanding of the pathogenesis, diagnosis, and treatment of fatigue associated with MS, despite the significant personal hardship and high socioeconomic cost. Hence, no interventions have amassed a sufficient degree of evidence, and neither the Food and Drug Administration (FDA) nor the European Medicines Agency (EMA) has approved any drugs [39]. The effects of vitamin D treatment over a six-month period were significant, according to the study of Achiron et al. [22]. Indeed, the FIS score was reduced by more than 40% in vitamin D-treated patients. Contrary to these findings, no changes were found in fatigue after vitamin D supplementation, as reported by two studies [29,31]. The negative results of these studies are consistent with previous research that found no correlation between serum 25(OH)D levels and scores on the physical subscale of the Multidimensional Fatigue Subscale [40]. This inconsistency could be due to the use of different measurement tools to estimate fatigue. The information offered here must be related to previous studies of fatigue interventions. Unfortunately, only some studies have shown a positive treatment influence on fatigue. In 195 MS patients, the impact of natalizumab on fatigue was evaluated in a practical situation. The study showed that the Fatigue Scale for Motor and Cognitive functioning decreased after 12 months of treatment but was neither blinded nor randomized [41]. In the same context, it was shown that the Modified Fatigue Impact Scale score significantly improved in 60 female MS patients treated for three months with ginseng, one of the herbal remedies having anti-fatigue qualities [42].

Several studies have reported an association of 25(OH)D levels with relapse risk [43]. Based on data from a prospective cohort of 145 patients with relapsing-remitting MS, most of whom were receiving immunomodulatory medication, the probability of recurrence may be expected to decrease by more than half with a 50 nmol/L increase in 25(OH)D levels [44]. Here, we noticed that supplementing vitamin D3 did not reduce the relapse rate in the majority of RCTs. These differences in results might be explained by differences in route of entry, type, and dosage of vitamin D metabolites used, and the genetic backgrounds of the MS patients. Although supplementation may have positive effects, particular levels should be taken into consideration as McLaughlin et al. found that arms receiving higher doses of vitamin D exhibited adverse changes in both ARR and EDSS [45]. In the same context, ARR and EDSS score changes were examined by Zheng et al., who found no positive effects of vitamin D as an adjunct therapy on either outcome [46].

The utilization of vitamin D supplements in the treatment of MS has been proposed based on the concept that serum 25(OH)D levels may be associated with the incidence and intensity of the disease course in MS patients. However, not all MS-related outcomes in the trials under evaluation appeared to be impacted by the rise in 25(OH)D levels. In contrast to other RCTs in this study, Camu et al. showed an improvement in EDSS score, and the 25(OH-D) levels at baseline were at the lower range of normal. Additionally, it has been demonstrated that high levels of 25(OH)D (>50 nmol/L) are related to decreased MS impairment as determined by the EDSS [29]. According to this review, it is still uncertain if vitamin D supplementation positively impacts lowering disability [47]. Similarly, a previous study demonstrated that EDSS score was not significantly different between the MS patients treated with a high or a low dose of vitamin D. Additionally, the controlled studies using 1α(OH)D3 or 1,25(OH)2D3 revealed evidence of a lack of efficacy [22,33].

In this systematic review, most of the included RCTs revealed a significant improvement in MRI brain lesion markers. Our results align with the EPIC cohort study that found that higher vitamin D serum levels were associated with the development of fewer T2 and gadolinium-enhancing brain MRI lesions [48]. It was suggested that the protective impact on new MRI lesions might only happen after more than six months of supplementation [49].

Dendritic cells and lymphocytes are two cell types that have nuclear receptors called VDRs [50]. Vitamin D exerts diverse effects on cellular processes such as proliferation, differentiation, and survival by binding to the VDR alongside the active vitamin D metabolite 1,25(OH2) D3, thereby influencing gene expression in chromosomal regions. [51]. Vitamin D has been demonstrated to directly suppress Th17 and Th1 differentiation and to induce Foxp3 regulatory (Treg) T cells [52]. T lymphocytes are directly controlled by vitamin D due to the expression of their VDR. Vitamin D supports T-cell development into Treg cells by reducing T-cell expression of the co-stimulatory receptors CD80 and CD86 and IL-12 production. Hence, the immunoregulatory impacts of vitamin D on adaptive immune responses appear to be conveyed through tolerogenic dendritic cells, regulatory Treg cells, and associated cytokines and growth factors [12]. This review revealed that TGF-β significantly increased in the vitamin D3-treated group. This finding is consistent with research conducted by Mahon et al., who discovered that MS patients who received vitamin D3 had higher serum levels of TGF-β [18]. One of the immunoregulatory cytokines made by Treg cells is TGF-β. It inhibits Th1 differentiation while encouraging additional Treg differentiation. When 1,25(OH2) D3 is administered in vitro to human skin Langerhans cells, these dendritic cells express TGF-β directly [53]. However, concentrations of various cytokines such as IFN-γ, IL-17A, IL-2, and IL-10 did not change statistically significantly in the vitamin D3 treatment group. These inconclusive findings could be attributed to the limitations of serum cytokine measurements compared to those obtained from the cerebrospinal fluid, stimulated leukocyte supernatants, or at the mRNA level from isolated leukocytes. A higher dose of vitamin D3 treatment could have been required to completely achieve the potential immunomodulatory effects. In their investigation, Burton et al. administered vitamin D levels up to 40,000 IU/day and found that treatment reduced T-cell reactivity and proliferation in the participants [54].

Even though further studies are required, this review emphasizes the importance of including baseline vitamin D as part of the evaluation in all future studies. Additionally, there was little risk of bias in most studies, and thus the validity is good. This updated review is more comprehensive, encompassing a broader range of MS symptoms and pathology. It features a more rigorous search methodology and bias assessment compared to previous reviews. While there may be similarities with some studies included in other reviews, the present study is more thorough and includes a detailed evaluation of the risk of bias.

While we have employed modern study design methodology, it is important to interpret our findings cautiously considering several limitations. Since the included trials were conducted in various locales, the exposure to the sun varied, making the comparison less accurate. The duration of the disease before the start of treatment differed between trials, and the timing of the vitamin D intervention may influence how well it works. While some studies just evaluated the results of the biomarkers, others also evaluated clinical endpoints such as relapse rates and disability scores. It is possible that the heterogeneity of outcomes might have affected the end-line comparisons, which could have prevented the possibility of conducting a meta-analysis.

## Conclusions

Administering vitamin D as a therapy for MS has shown encouraging results and warrants further exploration. This study highlights that high-dose vitamin D supplements can enhance physiological mechanisms, particularly in individuals with low baseline plasma levels, potentially leading to improved symptoms and reduced relapse rates. Despite these positive outcomes, the review underscores the necessity for more rigorous and extensive research to establish a definitive link between vitamin D supplementation and MS disease activity. Factors such as varying sun exposure, disease duration before treatment, and differences in outcome measurements across studies indicate the need for standardized approaches in future research. Moreover, understanding the optimal dosage, duration of treatment, and the specific subgroups of MS patients who may benefit the most from vitamin D supplementation is crucial. Future studies should also consider long-term safety and potential adverse effects to provide comprehensive guidelines for clinical practice. Overall, while vitamin D shows promise as a supplementary treatment for MS, conclusive evidence from well-designed clinical trials is essential to integrate this approach into standard care protocols fully.
